# A Case of *Scopulariopsis brevicaulis* Endocarditis with Mycotic Aneurysm in an Immunocompetent Host

**DOI:** 10.1155/2015/872871

**Published:** 2015-03-17

**Authors:** Kelly Cawcutt, Larry M. Baddour, Mary Burgess

**Affiliations:** ^1^Division of Infectious Diseases, Mayo Clinic, Rochester, MN 55905, USA; ^2^Division of Infectious Diseases and Myeloma Institute for Research and Therapy, UAMS, Little Rock, AR 72205, USA

## Abstract

*Scopulariopsis* is a genus of mold that is usually associated with onychomycosis and rarely causes complicated infection in immunocompetent persons. We describe a case of an immunocompetent 65-year-old male with a history of mitral valve repair with prosthetic ring placement who developed acute left posterior knee pain. Imaging showed a left popliteal artery aneurysm and thrombus, and further evaluation with transesophageal echocardiogram demonstrated two large, mobile mitral valve vegetations. He underwent debridement and replacement of the mitral valve, followed by debridement of the left popliteal artery with peroneal artery bypass. The intraoperative cultures grew *Scopulariopsis brevicaulis*. Due to the resistant nature of the organism, he was initially treated with combination antifungals including liposomal amphotericin B, caspofungin, and voriconazole and was continued on chronic suppression with posaconazole with no evidence of recurrence. *Scopulariopsis* is a rare cause of fungal endocarditis. Treatment of *Scopulariopsis* endocarditis is challenging and is not well understood due to its rarity.

## 1. Introduction

Fungal endocarditis is a rare disease comprising approximately 1–6% of all cases of endocarditis [1]. The incidence of fungal endocarditis has been increasing, likely secondary to advances in modern medicine, placing increasing numbers of patients at risk. Herein, we describe a rare cause of fungal endocarditis in an immunocompetent patient [2].

## 2. Case

A 65-year-old male marathon runner presented to a primary care physician with abrupt onset of posterior left knee pain that began two days before. There had been no injury associated with the pain. He denied systemic symptoms, including fever, chills, night sweats, or malaise. Past medical history included asymptomatic severe mitral valve regurgitation secondary to bileaflet mitral valve prolapse, for which he underwent robot-assisted minimally invasive mitral valve repair and prosthetic ring placement approximately 6 months prior to presentation. There was also a history of hypertension and hyperlipidemia. He lived in the central United States with his wife and was employed as an attorney. He consumed approximately two alcoholic drinks per night, was a never smoker, and had no history of intravenous drug use. There were no other significant exposures or travel documented.

The patient recovered from his mitral valve repair, had stopped anticoagulation after 6 weeks, and had returned to walking and/or running 3–5 miles per day. A transthoracic echocardiogram that was done approximately 2 months postoperatively demonstrated a normal functioning valve, with only trace regurgitation.

He was initially treated with symptomatic pain relief with hydrocodone-acetaminophen but had no improvement in the left popliteal fossa pain and was therefore referred to an orthopedic provider. A plain radiograph demonstrated Baker's cyst and he was treated with a cortisone injection. Due to persistence of symptoms, he was subsequently evaluated for possible deep venous thrombosis via venous ultrasound which indicated a left popliteal thrombus and aneurysm. As a result of this finding, he was admitted to an outside hospital for further evaluation.

At the outside facility, he underwent MRI of the left knee, CT angiogram with bilateral extremity runoff, and transthoracic echocardiogram. These studies revealed left popliteal aneurysm with occlusive eccentric thrombus and incomplete clot of a right renal artery branch. The following day, a transthoracic echocardiogram was done and showed a highly mobile mass attached to the anterior mitral valve leaflet, measuring approximately 1.5 cm.

Due to the abnormal findings, he was transferred to our facility for further evaluation and management. On admission, the patient appeared well. Temperature was 36.8°C, blood pressure was 123/48, heart rate was 85 beats per minute, respiratory rate was 16 breaths per minute, and peripheral oxygen saturations were 98% on room air. A 1/6 holosystolic murmur heard best over the mitral valve was auscultated; the remainder of the physical examination was normal. There was no mention of onychomycosis. His laboratory evaluation demonstrated mild anemia (11.4 g/dL; reference 13.5–17.5 g/dL), elevated erythrocyte sedimentation rate (60 mm/1 hour; reference 0–22 mm/1 hour), and elevated C-reactive protein (75.9 mg/L; reference < 8.0 mg/L). The peripheral white blood cell count and differential, platelet count, INR, and prothrombin time were all normal.

**Figure 1:**
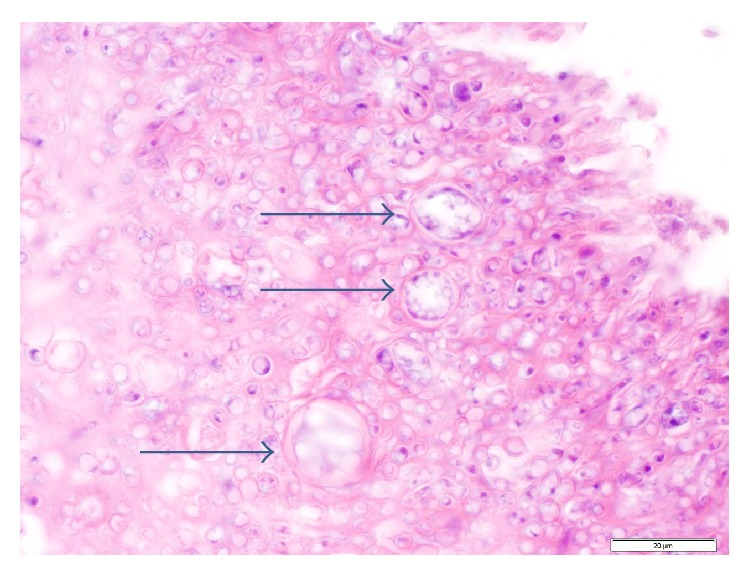
HE stain (100x) demonstrating bulbous forms.

**Figure 2:**
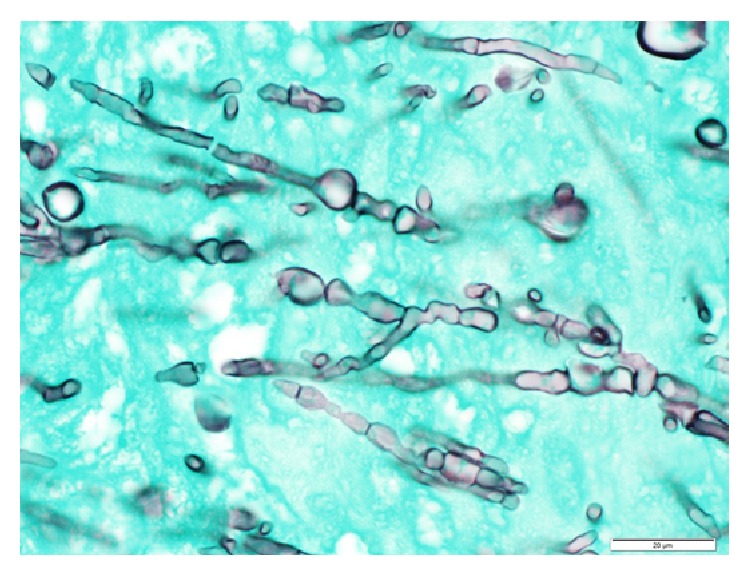
GMS stain (100x) demonstrating fungal forms.

The patient underwent transesophageal echocardiogram (TEE) which revealed two highly mobile masses attached to the mitral valve with the largest mass measuring approximately 1 cm by 3 cm. The patient underwent surgery the following day. Intraoperatively, the mitral valve had firm, white vegetations with destruction of both anterior and posterior leaflets. Samples were taken and sent immediately to pathology for review. The preliminary frozen pathology demonstrated fungal elements (Figures 1 and 2). He underwent aggressive debridement and mitral valve replacement with a porcine valve. The patient was started on intravenous liposomal amphotericin B 5 mg/kg/day intraoperatively and caspofungin 50 mg daily was added the next day for empiric treatment while waiting identification of the organism. Final pathology described active native valve endocarditis with fungal organisms involving the myxomatous mitral valve. The mitral valve tissue was cultured on inhibitory mold agar and ultimately grew a* Scopulariopsis* species with no other microbial growth, including negative bacterial and mycobacterial cultures. Fungal blood cultures were obtained but were negative for growth. Due to the severity of the disease and highly resistant nature of the organism, oral voriconazole dosed at 300 mg twice daily was also added to the antifungal regimen.

The day following mitral valve debridement and replacement, vascular medicine and vascular surgery were consulted regarding the suspected mycotic aneurysm and thrombus of the left popliteal artery. CT angiogram with lower extremity runoff was repeated and showed the left popliteal artery mycotic aneurysm and thrombus, as well as bilateral renal infarcts. Ten days after mitral valve surgery, he underwent resection of the popliteal artery and peroneal artery bypass. The pathologic examination revealed GMS-positive staining hyphae, and inhibitory mold agar cultures again grew a pure culture of* Scopulariopsis* species.

The* Scopulariopsis* species from the cultures was ultimately identified by combined phenotypic characterization and DNA sequencing as* Scopulariopsis brevicaulis*. Phenotypically, the organism was a tan powdery colony and microscopically demonstrated branching annellides with chaining, round conidia with a truncated base consistent with* Scopulariopsis brevicaulis*. From a sequencing standpoint, D2 ribosomal sequencing was utilized with the top 3 matches being a greater than 95% match (100%, 99.31%, and 95.45%) to* Scopulariopsis* species in the Mayo Fungal Library. Antifungal susceptibilities were completed utilizing broth dilution as per recommended guidelines from the Clinical and Laboratory Standards Institute and were consistent with those reported in the literature: amphotericin 2 *µ*g/mL, caspofungin 1 *µ*g/mL, micafungin 0.125 *µ*g/mL, anidulafungin 4 *µ*g/mL, posaconazole 2 *µ*g/mL, voriconazole 8 *µ*g/mL, itraconazole 2 *µ*g/mL, and terbinafine 0.5 *µ*g/mL [3].

Liposomal amphotericin was discontinued after 18 days due to acute renal injury, and caspofungin and oral voriconazole therapy were continued. He completed 8 weeks of combination therapy and was continued on oral voriconazole for chronic suppression. Voriconazole was chosen despite susceptibility data given reported clinical efficacy combined with lack of correlation between the susceptibility data and patient outcomes [4]. He unfortunately developed peripheral neuropathy, suspected by his local neurologist to be secondary to the voriconazole, and he was transitioned to delayed-release oral posaconazole dosed at 100 mg daily for chronic suppression [5, 6]. Posaconazole levels were followed and remained therapeutic with levels >1.0 mcg/mL. The inflammatory markers normalized at 6 months. Follow-up TEEs at 3 months and 7 months postoperatively did not show recurrence of endocarditis. Further, after cessation of the voriconazole he reported improvement, but not full resolution, of his neuropathy symptoms.

## 3. Discussion

Although fungal endocarditis is rare, clinicians must be aware of the differences in presentation and risk factors associated with it as compared to bacterial endocarditis. Risk factors associated with fungal endocarditis include native valvular disease, heart surgery (with prosthetic devices or heart valve replacement), intravascular devices, immunosuppression or immunocompromised states, broad-spectrum antibiotic use, and intravenous substance abuse [2].

The most common cause of fungal endocarditis is* Candida *species, with* C. albicans *being the most prevalent.* Candida *species are responsible for up to two-thirds of cases of fungal endocarditis followed by* Aspergillus *species which comprises another 25%. Therefore, only a very small percentage of fungal endocarditis is caused by other pathogens, such as* Scopulariopsis *species as seen in this case [2, 7].

Few cases of endocarditis due to* Scopulariopsis brevicaulis* have been reported in the literature [2, 7–10].* Scopulariopsis *species are filamentous fungi that are ubiquitous in the environment and are a known cause of onchomycosis; however, they rarely cause deep infections, such as endocarditis. Of all the* Scopulariopsis *species,* Scopulariopsis brevicaulis *is the most common organism seen clinically [4, 9]. As with other causes of fungal endocarditis, embolic phenomenon is common and has been reported in the majority of published cases of endocarditis secondary to* Scopulariopsis *species [2, 9]. Diagnostically, blood culture data has a limited role in diagnosis as less than 50% of fungal endocarditis cases demonstrate positive culture results, and of the positive cultures yeast is much more likely to be isolated than molds [1, 2]. Thus, the diagnosis is often based on a combination of histopathologic features and microbiologic data including culture and molecular testing but can be difficult initially, as many fungi that cause endocarditis may look very similar under the microscope. For instance,* Scopulariopsis *species may demonstrate hyaline or septate hyphae which could potentially appear very similar to* Aspergillus*,* Mucor*,* Scedosporium*, or* Candida *species; therefore, identification of the etiological agent is necessary for providing appropriate antifungal management of such patients [4, 8, 10].

Treatment for fungal endocarditis often includes both medical and surgical management, which has been recommended for invasive* Scopulariopsis *species infections by recent European guidelines [11]. A minimum of 6 weeks of antifungal therapy is recommended, and providers must consider subsequent suppressive therapy [1, 2]. Historically, amphotericin B has been the primary agent used in combination with newer agents, such as the echinocandins, voriconazole, and posaconazole [2]. Unfortunately,* Scopulariopsis *species demonstrate elevated minimum inhibitory concentration (MIC) breakpoints to currently available antifungal agents during in vitro testing, suggestive of resistance. However, due to the rarity of invasive disease, MIC data is too sparse to determine true resistance thresholds and correlation of susceptibility data with clinical outcomes is not well understood [2, 4]. In a recent study, of all classes of antifungal agents, echinocandins demonstrated the highest level of in vitro activity. Voriconazole, posaconazole, and terbinafine showed limited activity, and isolates were rarely susceptible to amphotericin B [4]. Given the rarity of such infections, optimal therapy remains undefined [11]. Combination therapy with amphotericin B, an echinocandin, and/or voriconazole or posaconazole has been utilized and may be reasonable despite the high levels of in vitro resistance as synergy has been reported for some isolates [4, 9, 10]. Despite lack of susceptibility demonstrated in vitro, some success has been seen clinically [4, 8].

Finally, outcomes of patients with fungal endocarditis vary, depending on the organism and the underlying comorbidities of the patient. Acute survival for all cases of fungal endocarditis approaches 50%. However, of those who survive, 30–50% will develop subsequent endocarditis relapse [2, 7]. Unfortunately, deep infections caused by* Scopulariopsis *species often portend a poor prognosis, in part secondary to the highly resistant nature of the organism.

## 4. Conclusion

Fungal endocarditis remains a rare clinical entity; however, with increasing numbers of patients undergoing cardiac surgery with placement of prosthetic valves and other valvular devices, the incidence is reportedly rising. Symptoms secondary to embolic events are a typical presentation.* Scopulariopsis* endocarditis is an uncommon cause of fungal endocarditis and is difficult to treat. This case illustrates the need for a heightened level of awareness among clinicians for fungal endocarditis in immunocompetent patients who have undergone valve replacements or repair.
